# Detection of helicobacter pylori in benign laryngeal lesions by polymerase chain reaction: a cross sectional study

**DOI:** 10.1186/1750-9378-7-10

**Published:** 2012-04-19

**Authors:** Farzad Izadi, Aslan Ahmadi, Shadi Ghourchian, Ahmad Daneshi, Faramarz Memari, Ehsan Khadivi, Shabahang Mohammadi

**Affiliations:** 1 Head and Neck Research Center, Hazrat Rasoul Akram Hospital, Tehran University of Medical Sciences, Tehran, Iran; 2 Medical student, Students’ Scientific Research Center (SSRC) of Tehran University of Medical Sciences, Tehran University of Medical Sciences, Tehran, Iran

**Keywords:** Helicobacter Pylori, Laryngeal lesion, Benign, In-house PCR

## Abstract

**Background:**

Although Helicobacter Pylori (HP) was detected in some cases of chronic laryngitis, the results were not confirmed by polymerase chain reaction (PCR). By this time, it has not been found in laryngeal lesions by in house PCR, the most sensitive method for detecting the genome tracks. Regarding the previous results and also few numbers of studies about the presence of HP in benign laryngeal lesions, specifically by PCR, we aimed to investigate the presence of HP in benign laryngeal lesions by in-house PCR.

**Methods:**

The samples were taken from 55 patients with benign laryngeal lesions and frozen in −20°C. One milliliter (ml) of lysis buffer was added to 100 mg (mg) of each sample and the tube was placed in 56°C overnight. Then DNA extraction was carried out.

**Results:**

To find HP DNA, in-house PCR was performed that revealed 5 positive results among 55 patients with benign laryngeal lesions. Of them, 3 were polyp, 1 was nodule and 1 was papilloma.

**Conclusion:**

Although the number of positive results was not a lot in this study, it was in contrast with previous studies which could not find any HP tracks in benign laryngeal lesions by other methods. More studies about the prevalence of HP in benign laryngeal lesions improve judging about the effect of this infection on benign laryngeal lesions.

## Background

Helicobacter pylori (HP) is one of the risk factors of adenocarcinoma. Thus, it was categorized as a carcinogen in 1994 by the World Health Organization (WHO) [1,2]. Recent studies have revealed that HP can be responsible for the pathogenesis of upper gastro-esophageal tract lesions such as oral cavity. It could be found in dental plaques and secretions of the salivary glands, even when gastric involvement was not detected [3-9].

HP was detected in some cases of chronic laryngitis (6/35) by rapid urease test [10]. However, it has not been confirmed by histology, tissue cultures, immunohistochemical methods or polymerase chain reaction (PCR) in benign laryngeal lesions [11,12]. Previous tests showed false positive or false negative results, regardless of the behavior of the lesion (malignant or benign) [11,13].

PCR is one of the most progressive methods in molecular biology and recombinant DNA technology. The technique is used to detect smallest particles in clinical specimens with a high specificity and sensitivity [14].

**Figure 1:**
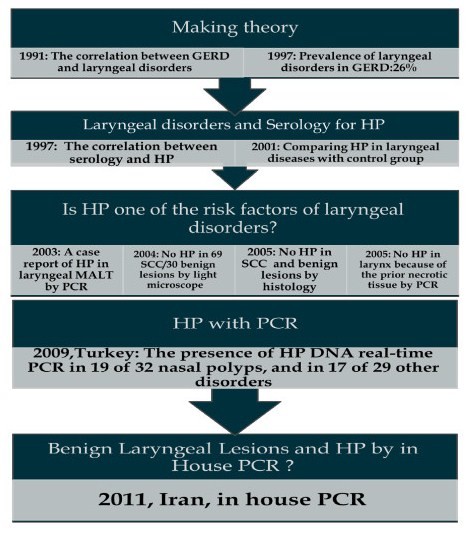
Different methods used to find helicobacter Pylori in laryngeal lesions, from past to now.

The last study performed in Turkey to investigate HP in laryngeal lesions by real-time PCR and in house PCR, revealed that 58.6 % of benign laryngeal lesions were infected with HP by real time PCR, but they didn’t detect HP by in house PCR [15] (Figure 1). Following that article and also the absence of other previous studies about the presence of HP in benign laryngeal lesions, specifically by PCR, we aimed to investigate the presence of HP in benign laryngeal lesions by in house PCR.

## Methods

### Ethical approval

The protocol of this study was approved by the institutional review board of the ENT research center of Rasul-e-Akram Hospital, Tehran University of Medical Sciences (TUMS). Before starting the investigation, verbal consent was established and the aim of the study was explained to all participants. Our patients did not agree to undergo endoscopy for investigation of HP in stomach.

To diagnose patients’ disorders, the specimens were obtained from laryngeal masses. It was used for our study, if the diagnosis was confirmed as a benign laryngeal lesion. The costs were supported by the institutional board.

### Study design and participants

This study was an observational cross-sectional study. Fifty five specimens with confirmed benign laryngeal lesion were evaluated for HP existence in the involved tissue. The patients with the symptoms of gastro-esophageal reflux disease (GERD) or positive history of using anti-HP drugs during the last month were excluded from the study.

Patients with GERD symptoms usually use some anti HP drugs even when the diagnosis is not confirmed with the gold standard. Regarding the ethical aspects of the study, we could not use 24-h PH monitoring (as the gold standard) or upper gastrointestinal tract endoscopy for the diagnosis. To make sure that the entered patients did not use any anti HP drugs, we examined the patients with GERD symptoms.

Any kinds of manipulations of intact and healthy laryngeal tissue can interfere with the vocal cords strength. In the other hand, designing the control group in our study should be based on the prevalence of the HP among intact laryngeal tissues. Regarding the illegal aspects of case–control study, we did not consider any control groups for our study. To compare the results with the frequency of HP in healthy tissues, we searched for other investigations despite the illegal entity of the method. No previously reported prevalence of HP in intact laryngeal tissue was found for comparison.

### Preparation of tissue samples

The tissue samples (2 × 2.5 mm of the laryngeal lesion) were extracted from 55 patients with benign laryngeal lesions in Rasul-e-Akram hospital. They were transferred to media in normal saline and frozen to –20°C.

### DNA extraction

A hundred mg of each sample was added to 1mililiter (mL) of lysis buffer (containing of 2 mg (mg)/mL, proteinase K) and the tube was placed in 56°C overnight. Then DNA extraction was carried out according to Phenol-Chloroform extraction method. It was kept in –20°C.

### Primers design

Two 20-base primers: upstream primer 1520–1522 nucleotide position (n.p.):5'-3' AATACACCAACGCCTCCAAG and downstream primer, 1935–1955 nucleotide position (n.p.): 5'-3' ATCTCAAGCTAACAGCCAAAA) were designed by primer 3 computer software. Analysis of primers by BLAST software showed that both primers had 100 % homology with HP genome and could identify different strains. These primers targeted CagA gene of HP (full genome sequence NCBI: FJ42821501 associated number) and amplified a 496-pb fragment.

### In-house PCR optimization

PCR optimization was performed using various amounts of magnesium chloride 2 (MgCl2), dNTPs, primers and different annealing temperatures using several dilution of template DNA. To amplify the fragments in each sample, 10 μl (1 μg) extracted DNA was added to a reaction mixture containing dNTPs, 200 μM; 10 pmol of each primer, MgCl2 1 μl; 50 mM and Taq DNA polymerase, 1U; (Ferments, Lithuania) in a total reaction volume of 25 μl. The samples were amplified for 40 cycles in an oil free automated thermal, verity of ABI, USA consisting of first denaturation at 94°C for 5 min, followed by cycles of denaturation at 94°C for 30 s, annealed at 50°C for 30 s and extension at 72°C for 40 s, with a final incubation at 72°C for 5 min. The PCR products were separated on 3 % agarose gel and stained with 3 μg/ml ethidium bromide solution. The stained gel was photo grated using gel documentation instrument with a digital imaging camera system (UV tech, Germany).

### Check list and analysis

To gather patients’ data, the drug history specially any anti HP drugs, and the results of their own biopsies and in-house PCR, were entered to a self-designed check list. We used SPSS software version17 for analysis.

To describe the prevalence of different qualitative variables descriptive indexes were used. Also mean and standard deviation (SD) were used to present quantitative variables.

## Results

Of 55 patients, 33 (60 %) were male. The mean age was 22 ± 10.57 years old with the range of 5 to 55 years of age. None of the patients used any drugs during the last month. None of them had any symptoms of GERD. Also the patients didn’t undergo any upper gastroesophageal tract manipulations, previously. Polyp was the most prevalent disorder among our patients (38.18 %). The prevalence of positive results among each group of patients is shown in Table 1.

**Table 1:** The prevalence of positive results among 55 patients with benign laryngeal lesions

Polyp: 21 (38.18 %)	Nodule: 14 (25.45 %)	Papilomatosis: 20 (36.36 %)
Positive results: 3 (14.28 %)	Positive results: 1(7.14 %)	Positive results: 1 (5 %)

In-house PCR of HP DNA revealed positive results in 5 of 55 patients (9.09 %). Fifty five specimens included 21polyps (38.18 %), 14 nodules (25.45 %) and 20 cases of papillomatosis (36.36 %). Of the specimens which were positive for HP, 3 (60 %) were polyp, 1 (20 %) was nodule and 1 (20 %) was papilloma.

## Discussion

HP was identified as a risk factor for lots of malignancies. About half of the persons around the world are infected by HP [16,17].

If HP involves the parts surrounding the lower esophageal sphincter, the probability of GERD increases. GERD increases the risk of malignancies in upper parts of gastro-esophageal tract. Of the organs located in the tract, larynx is prone to laryngeal carcinoma [18,19]. Also HP can be one of the risk factors of nasal polyp by causing chronic inflammation [11,20-24].

Among the studies investigated the relationships between GERD and laryngeal injuries, Koufman showed that 40–60 % of patients with voice disorders were presented by reflux symptoms [25]. Batch found the prevalence of 63 % of esophagitis in patients with laryngitis [26]. Paterson detected GERD in 26 % of patients with laryngeal disorders [27]. Because HP infection in stomach and so its distribution to esophagus was prevalent among the Iranian people [28], we didn’t investigate HP in esophagus in our study. In addition, our patients didn’t agree to undergo endoscopy. But HP infection was not usual in larynx.

In 2004, Ahmet Kizilay, et al. refused the presence of HP in their specimens from 69 cases with squamus cell carcinoma (SCC) and 30 patients with non-neoplastic diseases of larynx by using light microscope [29]. Also in the same year, Nihat Akbayir, et al. didn’t find HP in the specimens from patients with laryngeal SCC (50) by means of histology [11].

Furthermore, the presence of HP in malignant laryngeal lesions (by using immunohistochemical methods and light microscope) was compared with the presence of HP in benign laryngeal lesions (by histopathology) [11].

Prior to invasive methods, some investigators evaluated the serologic changes in HP infected patients and the prevalence of laryngeal cancer. Their results were in conflict. Some studies revealed significant differences between the prevalence of HP in patients with laryngeal cancer and the control group [29-31]. The results of the correlation between HP existence and laryngeal tumors were different [32]. In the other hand, some studies revealed that HP was not detected in malignant laryngeal lesions by immunohistochemical methods but there were some positive results by histological evaluations. Also they did not find HP tracks in specimens with benign laryngeal lesions in that study. The authors supposed that spontaneous eradication of HP in the malignant tissues which are surrounded by atrophic areas is the reason of their findings but this theory could not explain the absence of HP in non-atrophic tissues [11]. Some articles revealed a high prevalence of HP in patients with squamous cell carcinoma (SCC) of the larynx by serology [30,33,34].

According to our search, except a case report in which the diagnosis of HP was confirmed by PCR in a patient with laryngeal MALT lymphoma [15,35], there was no specific method used for confirming HP in laryngeal cancers without false positive result [36]. PCR was used in a study in Turkey that studied 32 patients with nasal polyps, 9 patients with SCC, 5 patients with chronic inflammation, 8 cases with laryngeal nodules, 2 cases with laryngeal papillomatosis, 2 with laryngeal polyps, one with laryngeal web, one with laryngeal dysplasia, and one with laryngeal hemangioma. By using real-time PCR, they found HP DNA in 19 of 32 nasal polyps, and in 17 of 29 laryngeal samples with other disorders [15]. Also cag A of HP was found in 30 % of tonsil and adenoid tissues by real-time PCR and in 24.6 % of the same tissues by in house PCR [24,37] (Table 1).

By our investigation, according to the high specificity and sensitivity of in-house PCR, we found HP in the patients with benign laryngeal lesions including polyp, nodule and papilloma.

Although the number of cases in this study was more than other previous studies, most of our patients could not be entered following the usage of anti HP drugs. Most of our patients with papiloma used omeprazole (as a self therapy) to relieve their upper gastrointestinal symptoms. Thus we missed lots of our cases for this study due to the high prevalence of anti HP drugs consumers among our patients. We excluded patients with GERD symptoms, but because of logical aspects, we could not use 24-h PH monitoring or endoscopy to confirm the diagnosis.

In our study the samples should be sent for pathology and genetic studies. Some of the excised lesions were too small to be divided to two parts and thus we could not investigate HP genomes in some small species.

## Conclusion

Although the number of our positive results was not a lot, it was in contrast with previous studies which could not find any HP tracks in benign laryngeal lesions by other methods. More studies about the prevalence of HP in benign laryngeal lesions improve judging about the casualty of this infection in benign laryngeal lesions.

## Abbreviations

HP = helicobacter; PCR = polymerase chain reaction; SD = standard deviation.

## Misc

This study was performed in Head and Neck Research Center, Hazrat Rasoul Akram Hospital, Tehran University of Medical Sciences, Tehran, Iran

## Competing interests

The authors declare that they have no competing interests.

## Authors’ contributions

FI, AD, SM, FM, AA: study concept and design; acquisition, doing the surgeries, revising the article. EK: Laboratory studies, doing PCR, revising the article. SG: Acquisition, analysis and interpretation of data, writing the manuscript, critical drafting of manuscript and revision of manuscript. All authors read and approved the final manuscript.
